# Contemporary management pathways in liver trauma: predominant and successful use of non-operative strategies at a high-volume tertiary center

**DOI:** 10.1007/s00423-026-04051-0

**Published:** 2026-04-30

**Authors:** Bruno Cirillo, Gioia Brachini, Roberto Cirocchi, Chiara Tranfaglia, Andrea Mingoli, Lina De Paola, Eleonora Lori, Salvatore Sorrenti, Antonio Pesce

**Affiliations:** 1 Department of Surgery, Sapienza University of Rome, Rome, Italy; 2 Department of Surgery, General Surgery, University of Perugia, Perugia, Italy; 3 Department of Anatomical, Histological, Forensic and Orthopedic Sciences, Sapienza University of Rome, Rome, Italy; 4 Department of Wellbeing, Health and Environmental Sustainability, Sapienza University of Rome, Rome, Italy; 5 Department of Surgery, Azienda Unità Sanitaria Locale Ferrara, University of Ferrara, Lagosanto, Ferrara Italy

**Keywords:** Liver trauma, Non-operative management, Angioembolisation, Shock Index, Injury Severity Score, ROC analysis, University trauma centre, Era analysis

## Abstract

**Background:**

Non-operative management (NOM) has become the default for haemodynamically stable liver trauma, yet pathway-level performance and bedside predictors of mortality warrant contemporary appraisal in University trauma centres.

**Methods:**

We conducted a single-centre retrospective cohort study of consecutive adults (≥ 18 years) with blunt or penetrating hepatic injury managed within a protocolised, physiology- and CT-guided pathway (1 Jan 2018–30 Apr 2025). The primary endpoint was in-hospital mortality. Secondary endpoints included NOM failure, liver-specific complications, and era-level outcomes (2018–2021 vs. 2022–2025). Independent predictors of mortality were assessed with multivariable logistic regression; discrimination was evaluated by ROC analysis.

**Results:**

Among 350 patients, NOM was initiated in 304 (86.9%); delayed conversion to laparotomy occurred in 15/304 (4.9%), including 5 after prior angioembolisation (IR in 32/350; 9.1%). Overall in-hospital mortality was 14.9% (52/350) and clustered in patients with physiological compromise and high global injury burden. Definitive management stratified patients into NOM and operative management cohorts, with lower mortality after definitive NOM than after operative management. In multivariable analysis, Shock Index (OR 2.71 per unit; *p* = 0.022), ISS (OR 1.05 per point; *p* < 0.001) and lactate (OR 1.17 per mmol/L; *p* = 0.031) independently increased the odds of death, whereas GCS was protective (OR 0.89 per point; *p* = 0.002); AAST high grade and era were not independently associated. Model discrimination was excellent (AUC 0.87; Youden-optimal sensitivity 0.92, specificity 0.74); Shock Index alone achieved AUC 0.75, while AAST grade provided modest separation (AUC 0.608). Secondary transfers (40.3%) incurred no survival penalty. Era comparison showed reduced operative exposure (21.5%→12.3%, OR 0.51, *p* = 0.024) and mortality (18.5%→10.3%, OR 0.51, *p* = 0.035) after formalising the pathway.

**Conclusions:**

In a university trauma centre, a protocolised physiology- and CT-guided pathway delivered high NOM utilisation with low failure and era-level gains. Short-term mortality was driven predominantly by physiology and global injury severity rather than anatomical grade. Multicentre prospective and implementation studies are warranted to mitigate residual biases and validate pragmatic bedside risk composites.

## Introduction

In abdominal trauma, hepatic injury remains one of the commonest solid-organ lesions and an important determinant of both early haemorrhagic death and later hepatobiliary morbidity. Over the past two decades, management has shifted decisively from routine laparotomy to non-operative management (NOM) as the standard approach in haemodynamically stable patients. In keeping with ATLS principles, this strategy is anchored to rapid contrast-enhanced computed tomography (CT), which provides definitive staging, while subsequent care is guided by damage-control resuscitation and mature trauma-centre pathways integrating imaging, bedside physiology, and timely escalation [1]. Across guidelines and large institutional cohorts, NOM success rates of approximately 90–97% are consistently reported in the absence of peritonitis, hollow viscus injury, or refractory shock, and these figures now represent the contemporary benchmark for quality appraisal [1–7]. Although anatomical grading remains essential for describing injury severity, grade alone neither dictates management nor reliably predicts mortality once physiology and associated injuries are considered. The 2018 American Association for the Surgery of Trauma Organ Injury Scale (OIS-AAST) update improved imaging–pathology concordance, but early decision-making remains physiology-centred: haemodynamic status, shock index, transfusion requirements, and response to resuscitation determine the initial branch point, while CT defines whether NOM should consist of observation alone or include interventional radiology, and when escalation is required [2]. In blunt trauma, the likelihood of laparotomy rises with grade, yet survival is more closely linked to global injury burden and physiological derangement; in penetrating trauma, mechanism and associated injuries often outweigh grade in determining operative need [2, 8, 9]. Comparable patterns have been reported in other solid-organ injuries, including blunt renal trauma, further supporting physiology- and imaging-driven pathways over grade alone [10]. CT has evolved from a triage tool into an instrument of risk stratification. The OIS-AAST 2018 revision facilitated more standardised reporting across centres [2], while multiphasic CT—combining arterial, portal venous, and delayed acquisitions—has improved detection of vascular and biliary lesions requiring escalation. Hepatic vascular injury (HVI), including active extravasation, pseudoaneurysm, arterioportal shunt, or contained vascular disruption, predicts resource utilisation and the likelihood of interventional radiology in contemporary cohorts [11]. Delayed-phase imaging may also reveal subtle HVI or bile leakage not evident on earlier phases, supporting its inclusion when suspicion persists [11–13]. Within CT-guided NOM, angioembolisation (AE) has become the principal adjunct for arterial contrast extravasation or convincing evidence of ongoing haemorrhage. Its purpose is not to replace surgery, but to extend the safe boundaries of NOM while preserving viable parenchyma. Comparative studies and systematic reviews suggest that segmental or super-selective AE is associated with fewer liver-related complications than main or proximal hepatic artery occlusion, which carries greater risks of bile leak, collections, and hepatic necrosis [14–17]. This is physiologically plausible, as selective embolisation better preserves downstream perfusion and collateral support [18, 19]. The later complication profile of NOM is heterogeneous. Bile leak and biloma are the commonest adverse events and are usually amenable to ERCP and drainage, whereas hepatic artery pseudoaneurysm and delayed haemorrhage are uncommon and often present insidiously, with falling haemoglobin rather than overt haemodynamic collapse [20]. These observations support triggered rather than routine surveillance imaging, guided by haemoglobin trends and clinical suspicion. System organisation has also contributed materially to improved outcomes. High-volume centres with formal trauma governance, explicit NOM and IR activation criteria, and close links with hepatobiliary and critical care services have reported lower mortality over time and very low mortality among patients successfully managed non-operatively [7, 9, 21]. Even where round-the-clock IR access is not universal, clearly defined escalation plans reduce variability and failure modes, underscoring that the pathway itself, rather than any single procedure, is the true intervention. Haemodynamically unstable patients in shock should not undergo CT; only stable or stabilised patients are candidates for rapid contrast-enhanced imaging, while physiological and biochemical markers such as shock indices, lactate, base deficit, and, potentially, admission hypokalaemia may refine bedside stratification [22, 23]. Against this background, high-volume university trauma centres are particularly well placed to evaluate integrated management pathways. We therefore conducted a contemporary retrospective analysis from a high-volume university tertiary trauma centre with widespread adoption of NOM and ready access to interventional radiology. The study aimed to describe patient characteristics, injury severity, and management patterns in adult blunt and penetrating liver trauma; to quantify outcomes, with in-hospital mortality as the primary endpoint and ICU admission, length of stay, and need for rescue surgery and/or angioembolisation as secondary endpoints; and to assess temporal changes across two treatment epochs, with particular focus on trends in NOM, operative management, IR utilisation, and mortality.

## Materials and methods

We conducted a retrospective, single-centre observational cohort study at the Emergency Department and Trauma Service of Policlinico Umberto I, a tertiary university hospital and regional referral centre in Rome, Italy, over the period 1 January 2018 to 30 April 2025. The study was performed in accordance with the Declaration of Helsinki. Given its retrospective, anonymised design, formal Institutional Review Board approval was waived. At admission, all patients provided written informed consent for clinical treatment and for the scientific use of their data. Eligible patients were all consecutive adults (≥ 18 years) presenting either by primary transport or by secondary inter-facility transfer who, at index admission, had a blunt or penetrating liver injury documented on contrast-enhanced computed tomography or confirmed intraoperatively. Exclusion criteria were age < 18 years, duplicate encounters, with only the index admission retained, and records lacking sufficient information to establish hepatic injury and in-hospital outcome. Inter-facility transfers were included provided that definitive care and outcome occurred at our centre. Transport pathway was classified a priori as primary transport, namely direct arrival from the scene, or secondary transport, namely transfer from peripheral hospitals to our hub under regional trauma-centralisation rules [1]. Mechanism of injury was derived from the medical record and categorised as road traffic collision, pedestrian or bicycle struck, accidental fall, fall from height, penetrating injury, or assault. Hepatic injury severity was graded according to the American Association for the Surgery of Trauma Organ Injury Scale (OIS-AAST), grades I–V, on the index CT, supplemented where necessary by operative findings [2]. Collected variables included age, sex, transport category, mechanism, Shock Index, Glasgow Coma Scale (GCS), lactate, Injury Severity Score (ISS), associated injuries, and non-hepatic surgical procedures performed during the index admission, including hollow-viscus repair or splenectomy. Within the NOM pathway, interventional radiology was considered an integral component rather than a departure from non-operative management. In haemodynamically stable patients, arteriography with or without angioembolisation was selectively undertaken when contrast-enhanced CT demonstrated arterial contrast extravasation. In keeping with contemporary management pathways and prior literature, interventional radiology was therefore treated as a selective adjunct to NOM, and its indications and complications were assessed accordingly. Secondary surgical haemostasis after an initial NOM strategy was reserved for persistent or recurrent haemodynamic instability, ongoing or delayed bleeding, failed haemostatic control after observation or interventional radiology, or associated abdominal injuries mandating laparotomy. Era was operationalised as an early versus a later period according to the prespecified temporal stratification of the cohort. Statistical analysis was performed using IBM SPSS Statistics version 28.0 (IBM Corp., Armonk, NY, USA). Continuous variables were summarised as mean ± standard deviation or median [interquartile range], according to distribution, assessed as appropriate with the Shapiro–Wilk and Levene tests. Categorical variables were expressed as n (%). Comparisons between groups employed Student’s t-test or the Mann–Whitney U test for continuous variables, and Pearson’s χ² or Fisher’s exact test for categorical variables. Independent predictors of the primary endpoint were examined by multivariable logistic regression, with candidate covariates selected a priori on clinical grounds and supplemented by variables showing *p* < 0.10 on bivariate screening. Multicollinearity was assessed by variance inflation factor, calibration by the Hosmer–Lemeshow test, and discrimination by receiver operating characteristic analysis with area under the curve and the Youden-optimal operating point. Results are reported as odds ratios with 95% confidence intervals [1, 11]. All tests were two-sided, and *p* < 0.05 was considered statistically significant. Analyses were performed on available cases, with observations containing missing data excluded only from the specific analysis concerned.

## Results

**Fig. 1:**
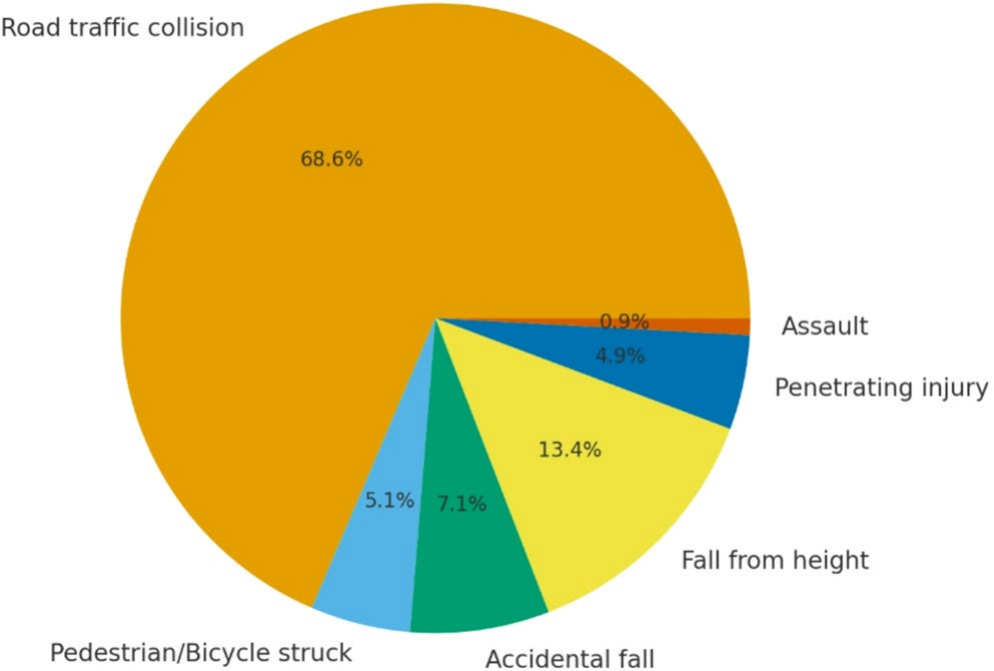
Mechanism of injury

**Fig. 2:**
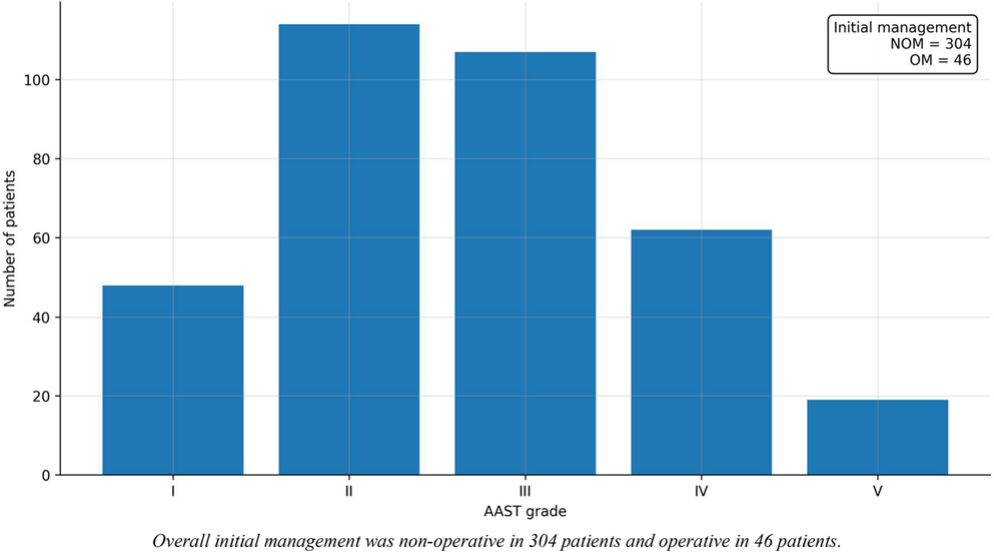
Distribution by AAST grade

Overall, 350 patients met the inclusion criteria and were included in the study population. The cohort comprised 245 men (70.0%) and the mean age was 39.6 years (median 35.0, range 19–80), consistent with a young-adult trauma population [3]. Blunt mechanisms predominated, with road traffic collision in 240 cases (68.6%), fall from height in 47 (13.4%), and accidental fall in 25 (7.1%); pedestrian or bicycle struck events accounted for 18 patients (5.1%), while penetrating injuries and assaults were less frequent at 17 (4.9%) and 3 (0.9%), respectively (Fig. 1). Injury severity by AAST showed 269 low-grade lesions (I–III; 76.9%) and 81 high-grade lesions (IV–V; 23.1%), a distribution that mirrors contemporary series from high-volume centres (Fig. 2). Primary transport comprised 209 (59.7%) cases, whereas secondary transfers accounted for 141 (40.3%) (Table 1).

**Table 1:** Demographics and clinical characteristics

Variable	Value
Number of patients	350
Male sex, n (%)	245 (70.0%)
Mean age, years	39.6
Median age, years	35.0
Mechanism of injury	
Road traffic collision	240
Pedestrian/Bicycle struck	18
Accidental fall	25
Fall from height	47
Penetrating injury	17
Assault	3
AAST grade	
I	48
II	114
III	107
IV	62
V	19
Low grade (I–III)	269
High grade (IV–V)	81
Length of stay, median [IQR], days	11.0 [7.0–21.5]
In-hospital mortality, n (%)	52 (14.9%)
Transport pathway	
Primary	209
Secondary	141

The proportion of high-grade (AAST IV–V) injuries was comparable across the two pathways, indicating that inter-facility transfers did not selectively concentrate the most severe hepatic lesions but rather operated within a regional centralization framework. In our setting, secondary transfer occurs explicitly under the trauma-center centralization rules, routing polytrauma patients from peripheral hospitals to a tertiary hub with round-the-clock CT, interventional radiology, and surgical capability [1, 3]. On Emergency Department arrival, patients demonstrated a moderately elevated heart rate (92.2 ± 19.9 bpm) and a reduced mean arterial pressure (101.7 ± 18.0 mmHg), consistent with variable degrees of early haemodynamic compromise. Overall injury burden was substantial, with an Injury Severity Score averaging 27.7 ± 15.9 (median 25.0, IQR 16.0–36.0). Admission haemoglobin was 11.7 ± 2.7 g/dL and mean Glasgow Coma Scale was 12.3 ± 4.6. ICU admission occurred in 30.9%; median ICU stay was 6.0 days [IQR 3.0–13.0]; median hospital length of stay was 11.0 days [IQR 7.0–21.5]. Operative management was undertaken in 61 patients (17.4%). Techniques were frequently combined; the most common components were direct haemostasis (32 cases, 52.5%) and hepatic packing (26, 42.6%), with parenchymal suturing used in 11 cases (18.0%) and one necessary left hepatectomy (1.6%). Among operated patients, AAST grades clustered in the higher spectrum (IV–V in 54.1%), with the following distribution: I 1 (1.6%), II 9 (14.8%), III 18 (29.5%), IV 26 (42.6%), V 7 (11.5%). Concomitant procedures reflected the polytrauma burden and included splenectomy (*n* = 19), bowel resection (*n* = 8), resuscitative thoracotomy (*n* = 13; clamshell thoracotomy *n* = 5), pelvic packing (*n* = 6), nephrectomy (*n* = 5), and cholecystectomy for gallbladder avulsion (*n* = 3). Fifteen additional patients arrived as secondary transfers having already undergone surgery at the referring hospital; these external procedures are reported descriptively but were not counted within our institutional operative caseload (Table 2).

**Table 2:** Operative management in liver trauma: techniques used, associated procedures, and AAST grade among operated patients

Category	Variable	Value
Operative technique	Hepatic packing	26 (42.6%)
Operative technique	Direct haemostasis (± topical agents)	32 (52.5%)
Operative technique	Parenchymal suturing	11 (18.0%)
Operative technique	Left hepatectomy	1 (1.6%)
Associated procedure	Splenectomy	19
Associated procedure	Bowel resection	8
Associated procedure	Resuscitative thoracotomy	13
Associated procedure	Clamshell thoracotomy	5
Associated procedure	Pelvic packing	6
Associated procedure	Nephrectomy	5
Associated procedure	Cholecystectomy for avulsion	3
AAST grade	I	1 (1.6%)
AAST grade	II	9 (14.8%)
AAST grade	III	18 (29.5%)
AAST grade	IV	26 (42.6%)
AAST grade	V	7 (11.5%)

*AAST* American Association for the Surgery of Trauma. Percentages refer to operated patients managed at our institution (*n* = 61). Associated procedures are reported as absolute numbers because more than one procedure could be performed in the same patient

Radiology-guided angioembolization was performed in 32/350 patients (9.1%) as an adjunct to non-operative management. By AAST grade, the IR subset comprised 2 grade II (6.2%), 11 grade III (34.4%), 13 grade IV (40.6%), and 6 grade V (18.8%). Expressed over the whole cohort, this corresponds to 0.6% (II), 3.1% (III), 3.7% (IV), and 1.7% (V) of all patients (Table 3).

**Table 3:** Interventional radiology (IR) cases by AAST grade among patients managed non-operatively (NOM)

AAST grade among IR	*n*	% of IR	% of total cohort
II	2	6.2%	0.6%
III	11	34.4%	3.1%
IV	13	40.6%	3.7%
V	6	18.8%	1.7%
Total	32	100%	9.1%

*IR* interventional radiology; *NOM* non-operative management;

Among patients initially managed non-operatively (*n* = 304), conversion to laparotomy occurred in 15 cases (4.9%), comprising 10 failures after strict observation (3.3% of the NOM population; 2.9% of the cohort) and 5 failures following IR-assisted NOM (1.6% of the NOM population; 1.4% of the cohort). Delayed operative haemostasis was undertaken for clinical or radiological failure of NOM, most commonly in the setting of haemodynamic deterioration, ongoing or delayed haemorrhage, or failed haemostatic control after IR when applicable. Within the embolized subgroup (*n* = 32), the IR→OR conversion rate was 15.6% (5/32), indicating that angioembolization achieved durable haemostatic control in the large majority of cases. Complementarily, 46 patients (13.1% of the cohort) underwent immediate operative management without a prior non-operative attempt. Collectively, these figures support the safety of a CT- and physiology-guided NOM strategy with selective angioembolization, with a limited conversion burden concentrated in a small subset (Table 4).

**Table 4:** Failure of non-operative management (NOM): overall, by pathway, and IR conversion (updated)

Metric	*n*	% of NOM	% of cohort
Initial NOM	304	—	86.9%
NOM failures (overall)	15	4.9%	4.3%
Strict NOM → OR	10	3.3%	2.9%
IR-assisted NOM → OR	5	1.6%	1.4%
IR within NOM	32	10.5%	9.1%
Immediate operative management (no prior NOM)	46	—	13.1%

IR → OR conversion among embolized cases: 5/32 (15.6%)

*NOM* non-operative management; *IR* interventional radiology (angioembolization); *OR* operating room. Percentages use the initial NOM population (n=304) or the overall cohort (n=350), as indicated

**Fig. 3:**
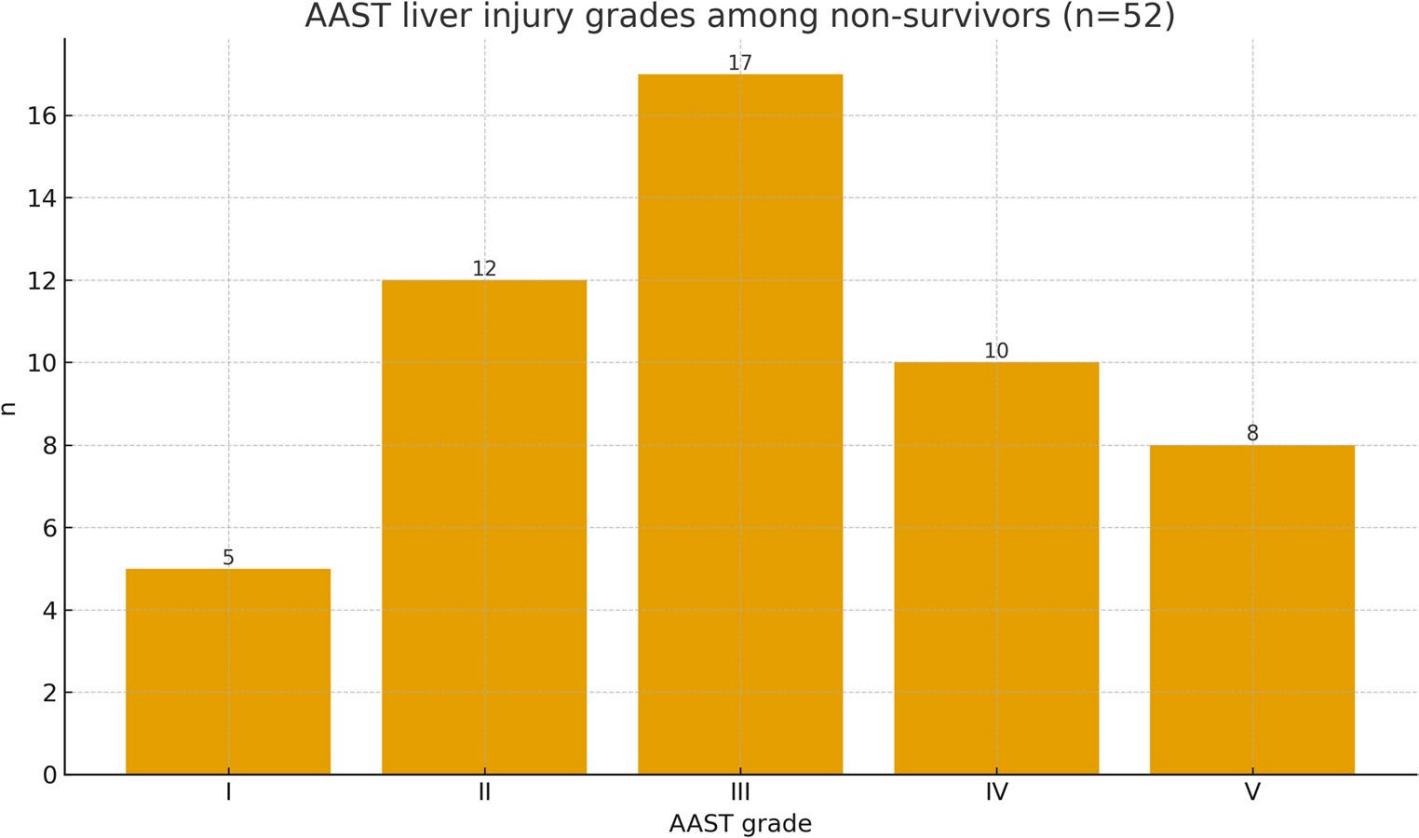
AAST grades among non-survivors (*n* = 52)

Among the 52 in-hospital deaths, decedents were predominantly male and in mid-life (mean age 50.7 years; median 49). Admission physiology signalled a substantial shock burden, with a mean systolic blood pressure of 110 mmHg and a mean heart rate of 95 bpm, together with marked anaemia on arrival (mean haemoglobin 9.0 g/dL; median 9.2 g/dL). The overall trauma burden was considerable, with an Injury Severity Score averaging 37.6 (median 34; maximum 75), fully consistent with multisystem injury. Referral pathways were broadly balanced between direct presentation and regional centralization, with 32 patients (61.5%) arriving by primary transport and 20 (38.5%) transferred inter-facility. Hepatic injury severity among non-survivors encompassed the full AAST spectrum yet clustered toward intermediate-to-high grades, as shown in Figure 3: grade I in 5 cases (9.6%), grade II in 12 (23.1%), grade III in 17 (32.7%), grade IV in 10 (19.2%), and grade V in 8 (15.4%). Accordingly, high-grade injuries (AAST IV–V) accounted for 18 of 52 deaths (34.6%), while AAST III contributed a further one-third of cases. The relatively high number of deaths observed in patients with AAST III injuries should not be interpreted as evidence that grade III liver injuries are more lethal than grade IV–V lesions. Rather, this finding is likely explained by the higher number of AAST III injuries in the overall cohort and by the fact that mortality in liver trauma is strongly influenced by haemodynamic status and overall trauma severity, rather than by liver injury grade alone. Operative exposure within the mortality subset was considerable: 23 decedents underwent surgery at our institution, and 4 additional patients arrived as secondary transfers already post-operative from spoke hospitals, reflecting both the acuity of presentation and the regional centralisation workflow. Taken together, the pattern depicts a mortality subgroup with marked physiological compromise at entry, high global injury severity, and a hepatic injury distribution concentrated in AAST III–V—culminating in fatal outcomes after both primary presentation and guideline-directed secondary centralisation.

**Fig. 4:**
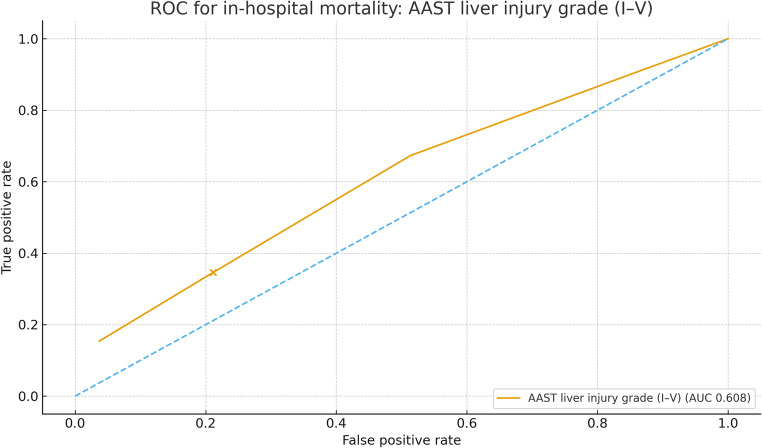
ROC curve for in-hospital mortality: AAST liver injury grade (I–V). The prespecified operating point at AAST ≥ IV is marked. Abbreviations: ROC, receiver operating characteristic; AUC, area under the curve

Definitive management stratified patients into mutually exclusive cohorts (NOM *n* = 289; OM *n* = 61), allowing a direct comparison of outcomes. Overall mortality was significantly lower in the NOM group than in OM (8.7% [25/289] vs. 37.7% [23/61]; *p* < 0.001), a difference that is consistent with the expected clinical selection of physiologically deranged and anatomically complex cases to surgery. Liver-specific morbidity likewise favored NOM: biloma occurred in 1.4% (4/289) under NOM versus 8.2% (5/61) after OM (*p* = 0.0099); delayed haemorrhage in 2.1% (6/289) under NOM—each managed conservatively—versus 13.1% (8/61) after OM (*p* = 0.0007); and hepatic abscess in 0.7% (2/289) under NOM—treated with interventional radiology drainage where indicated—versus 9.8% (6/61) after OM (*p* = 0.0005). Among patients initially treated non-operatively, 15/289 (5.2%) required delayed laparotomy (failure of NOM), and 3 of these 15 patients (20.0%) died after delayed surgery; notably, 5/15 failures (33.3%) had undergone prior interventional radiology before laparotomy. Taken together, these data indicate that, within a physiology- and imaging-guided pathway, definitive NOM is associated with lower mortality and fewer liver-specific complications than definitive OM, while acknowledging that between-group differences reflect indication bias rather than random allocation.Receiver–operating–characteristic analysis revealed heterogeneous discrimination across anatomical, physiological, and global severity indices. The AAST liver injury grade (I–V), analysed as an ordinal predictor, showed modest separation for in-hospital mortality (AUC 0.608; Fig. 4). For clinical interpretability, the prespecified threshold AAST ≥ IV yielded sensitivity 34.6%, specificity 78.9%, PPV 22.2%, NPV 87.4%, and Youden index 0.135. In contrast, non-figure analyses confirmed superior discrimination by global burden and physiology at presentation: the Injury Severity Score (ISS) achieved AUC 0.762, and the Shock Index (HR/SBP) AUC 0.805. Collectively, these findings indicate that while hepatic anatomical grading contributes complementary risk information, short-term mortality is more accurately captured by indices reflecting overall injury load and haemodynamic derangement on arrival.

Focusing on the contemporary window (1 January 2018–30 April 2025), the comparison between the eras 2018–2021 and 2022–2025 shows a clear contraction in operative exposure and crude mortality after adoption of a formalised, protocol-driven non-operative pathway. In 2018–2021, 195 patients were managed, with 42 laparotomies (21.5%) and 36 in-hospital deaths (18.5%); in 2022–2025, 155 patients were managed, with 19 operations (12.3%) and 16 deaths (10.3%). Accordingly, the proportion undergoing operative management fell by 9.2% points (from 21.5% to 12.3%), while mortality decreased by 8.2 points (from 18.5% to 10.3%). Expressed as odds ratios for the later versus earlier era, the odds of operative management were roughly halved (OR 0.51, 95% CI 0.28–0.92; *p* = 0.024), as were the odds of in-hospital death (OR 0.51, 95% CI 0.27–0.96; *p* = 0.035). Taken together, these temporal shifts are consistent with systematic implementation of a physiology- and CT-guided non-operative algorithm, whereby fewer patients proceeded to laparotomy and surgical candidates increasingly represented those with greater clinical severity or refractory haemorrhage (Table 5).

**Table 5:** Era analysis (2018–2021 vs. 2022–2025): operative management and mortality

Measure	2018–2021 (*n* = 195)	2022–2025 (*n* = 155)	Absolute difference (pp)	Odds ratio (95% CI); *p*
Operative management	42/195 (21.5%)	19/155 (12.3%)	−9.3	0.51 (0.28–0.92); *p* = 0.024
In-hospital mortality	36/195 (18.5%)	16/155 (10.3%)	−8.1	0.51 (0.27–0.96); *p* = 0.035

Odds ratio expressed as 2022–2025 vs. 2018–2021. Two-sided Fisher’s exact test. CI, confidence interval; ‘pp’ = percentage points

**Fig. 5:**
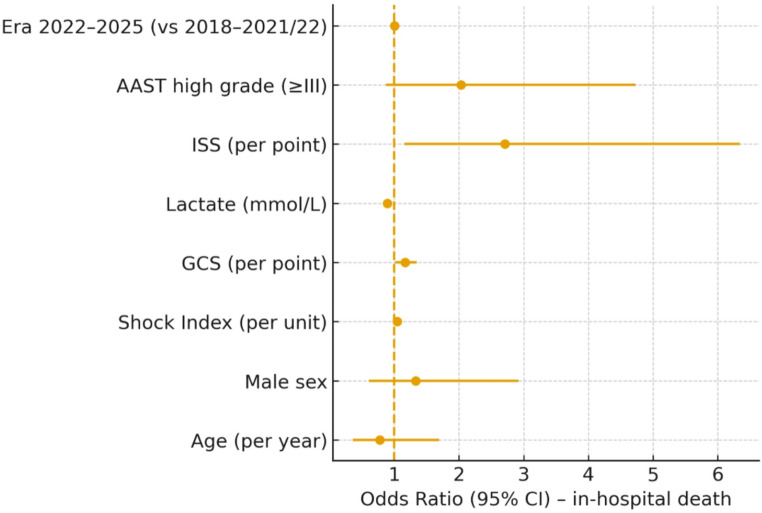
Forest plot

**Fig. 6:**
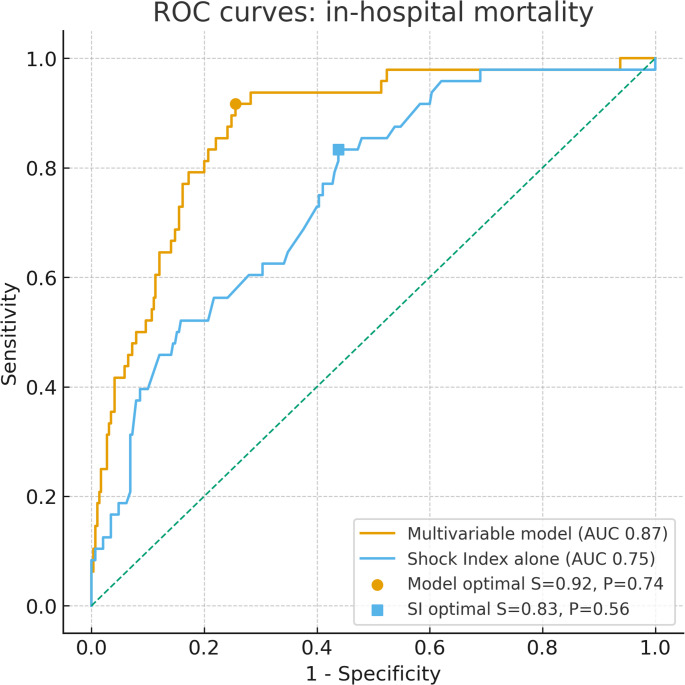
ROC curves comparing the multivariable model (AUC 0.87) versus Shock Index alone (AUC 0.75). Youden-optimal operating points are indicated. Abbreviations: ROC, receiver operating characteristic; AUC, area under the curve

We fitted a complete-case multivariable logistic regression (maximum-likelihood; *n* = 338, deaths = 48) including age, sex, Shock Index (heart rate/systolic blood pressure), GCS, lactate (mmol/L), ISS, AAST high grade (≥ III vs. I–II), and era (2022–2025 vs. 2018–2021/22). After adjustment, higher Shock Index (OR 2.71, 95% CI 1.16–6.34; *p* = 0.022), higher ISS (OR 1.05 per point, 95% CI 1.02–1.07; *p* < 0.001) and higher lactate (OR 1.17 per mmol/L, 95% CI 1.01–1.35; *p* = 0.031) were associated with increased odds of in-hospital mortality, whereas higher GCS was protective (OR 0.89 per point, 95% CI 0.83–0.96; *p* = 0.002); age (OR 1.01, 95% CI 0.99–1.03; *p* = 0.464), male sex (OR 2.03, 95% CI 0.87–4.73; *p* = 0.101), AAST high grade (OR 1.33, 95% CI 0.61–2.92; *p* = 0.477) and era (OR 0.77, 95% CI 0.35–1.69; *p* = 0.521) were not independently associated. The full adjusted estimates are reported in Table 6 and displayed as a forest plot in Fig. 5. Model discrimination was good (AUC 0.87); the Youden-optimal probability threshold was 0.12, yielding sensitivity 0.92 and specificity 0.74, with the ROC curve shown in Fig. 6. For context, Shock Index alone achieved an AUC of 0.75 with an optimal threshold of 0.85 (sensitivity 0.83, specificity 0.56).

**Table 6:** Adjusted Odds Ratios (95% CI, *p*-value)

Variable	OR	95% CI	*p*-value
Age (per year)	1.01	0.99–1.03	0.464
Male sex	2.03	0.87–4.73	0.101
Shock Index (per unit)	2.71	1.16–6.34	0.022
GCS (per point)	0.89	0.83–0.96	0.002
Lactate (mmol/L)	1.17	1.01–1.35	0.031
ISS (per point)	1.05	1.02–1.07	< 0.001
AAST high grade (≥ III)	1.33	0.61–2.92	0.477
Era 2022–2025 vs. 2018–2021	0.77	0.35–1.69	0.521

*OR* odds ratio; *CI* confidence interval; *GCS* Glasgow Coma Scale; *ISS* Injury Severity Score; *AAST* American Association for the Surgery of Trauma

## Discussion

In this contemporary single-centre cohort (*n* = 350) from a high-volume university trauma centre, three findings emerge directly and consistently from our data and frame the clinical message of the study. First, a physiology- and CT-guided pathway enabled initial non-operative management (NOM) in 86.9%, complemented by selective angioembolization (AE) in 9.1%, with a limited conversion burden (strict observation→OR 3.3%; IR-assisted NOM→OR 1.6%) (Table 4, Table 2). These proportions held across a broad case-mix typical of an urban tertiary hub—70.0% male, mean age ~ 40 years, and road traffic collision as the dominant mechanism—spanning the full AAST spectrum (I–V), which underscores the feasibility of a protocolised NOM paradigm in routine practice [1, 2]. Second, in-hospital mortality—our primary endpoint—was 14.9% overall but diverged sharply by definitive strategy (8.7% with NOM vs. 37.7% with OM; *p* < 0.001), with liver-specific morbidity likewise favouring NOM (biloma 1.4% vs. 8.2%; delayed haemorrhage 2.1% vs. 13.1%; hepatic abscess 0.7% vs. 9.8%; all *p* < 0.01) (Table 6). These between-group differences should be interpreted cautiously, given the inherent indication bias of observational trauma cohorts, in which operative management is preferentially reserved for patients with greater physiological derangement and more complex injury patterns. This pattern persisted despite inevitable indication bias toward surgery in more deranged or anatomically complex cases [3, 4]. Third, at system level, formal pathway implementation coincided with a contraction in operative exposure (21.5%→12.3%) and crude mortality (18.5%→10.3%) when comparing 2018–2021 with 2022–2025 (OR 0.51 for each; *p* = 0.024 and *p* = 0.035), suggesting reproducible gains attributable to consistent imaging, explicit escalation triggers, and reliable access to IR [5, 6]. The determinants of death in our series clarify why a NOM-first strategy anchored to physiology is both safe and rational. In multivariable analysis, Shock Index (OR 2.71), ISS (OR 1.05 per point), and lactate (OR 1.17 per mmol/L) independently increased the odds of in-hospital mortality, whereas GCS was protective (OR 0.89 per point); age, sex, AAST ≥ III, and era were not independently associated after adjustment. This mirrors the clinical reality of polytrauma: global injury burden and haemodynamic derangement at the bedside—rather than isolated anatomical grade—drive early outcomes once case-mix is accounted for [8, 9]. The profile of non-survivors in our cohort (median ISS ~ 41, physiological compromise on arrival, AAST clustering in III–V) is consistent with this interpretation [10]. Our discrimination analyses reinforce these points and translate them to practice. The multivariable model achieved AUC 0.87 with a Youden-optimal probability threshold of 0.12 (sensitivity 0.92, specificity 0.74), offering actionable operating characteristics for risk stratification (Fig. 6) [1]. Shock Index alone performed substantially better than anatomy (AUC 0.75), whereas AAST grade analysed ordinally showed modest separation (AUC 0.608); a pragmatic bedside cut at AAST ≥ IV traded acceptable specificity for low sensitivity (34.6%) (Fig. 4) [2, 8]. Practically, these curves argue for physiology-centred triage supplemented by global severity scales, using grade to describe and to guide targeted imaging or IR, but not as a solitary trigger for escalation [5, 7]. Within CT-guided NOM, our utilisation of multiphasic CT and selective, super-selective AE (IR in 9.1%; IR→OR 15.6%) exemplifies imaging as a risk-stratification tool rather than a mere triage gateway. Protocols incorporating arterial, portal-venous, and delayed phases improved detection of hepatic vascular injury (HVI)—active extravasation, pseudoaneurysm, arterioportal shunt, contained disruption—which, when present, justified AE and clarified who could be observed safely [11, 12]. The emphasis on segmental/super-selective embolization aligns with comparative and review data showing fewer bile-leaks, collections, and necrosis than proximal/main hepatic artery occlusion [14, 15]. The physiological rationale is coherent: limiting devascularisation while preserving portal inflow helps maintain the hepatic perfusion index in already-injured parenchyma [16, 17]. Reports of low clinically significant necrosis after selective AE and the occasional role of delayed/functional imaging in selected scenarios are consistent with this approach [18, 23]. The behaviour of hepatic artery pseudoaneurysm (HAPA) and delayed haemorrhage in our series—uncommon, often heralded by haemoglobin drift, and manageable with targeted AE when angiographically demonstrable—supports triggered re-imaging rather than routine surveillance for all NOM patients [19, 20]. System design appears pivotal. Our region’s centralisation rules facilitated timely secondary transfers without simply concentrating the most extreme grades at the hub; rather, they equalised access to round-the-clock CT, IR, and operative capability [7]. The era-level reductions in laparotomy and crude mortality mirror reports linking outcomes to formal governance, standardised IR activation criteria, and time-bound escalation thresholds [9, 21]. Notably, the era covariate did not retain an independent association with mortality after adjustment, implying that the causal lever is what the pathway does—rapid diagnostics, physiology-led decisions, judicious AE—more than calendar time per se [24]. This distinction matters for implementation: replicating process rules (who/when/how to image and escalate) is more likely to reproduce gains than importing equipment or expertise in isolation [25, 26]. Clinical implications follow directly from our results. First, early bedside triage should privilege physiology (Shock Index, GCS) and global severity (ISS) alongside CT; anatomical grade should not be used in isolation to govern escalation [2, 9]. Second, AE should remain selective and super-selective, guided by multiphasic CT (including a delayed phase when suspicion of HVI or occult bile leak persists), while surveillance imaging should be triggered by clinical/laboratory cues rather than performed routinely in all NOM patients [11, 14]. Third, programme performance hinges on predefined triggers and time-to-action metrics; where after-hours IR coverage is uneven, explicit stabilisation, imaging, and escalation plans constitute the actual intervention [7, 21]. Finally, simple biochemical screens may complement imaging at the bedside—e.g., stress-axis hypokalaemia or a pragmatic GLU/K ratio as coarse risk signals—within physiology-first algorithms [22]. This study has limitations. Its retrospective, single-centre design invites residual confounding and selection/indication bias, particularly in comparisons between definitive NOM and OM despite transparent reporting of conversions and IR utilisation [1]. Some variables were affected by missingness and were handled via complete-case modelling; after-hours IR was not standardised beyond institutional practice; and long-term hepatobiliary outcomes were not captured systematically [11]. Strengths include a consecutive tertiary-centre cohort across two epochs, consistent pathway implementation, comprehensive reporting of NOM, AE, conversions, complications, ICU and LOS, and analytic triangulation using multivariable modelling with ROC-based appraisal of discrimination [5, 6]. In summary, within a protocolised NOM pathway reinforced by selective, super-selective AE, most adults with blunt or penetrating liver injury can avoid laparotomy, and short-term mortality is chiefly determined by physiology and global injury severity rather than anatomical grade alone [2, 8]. The era-level reductions we observed in operative exposure and crude mortality appear mediated by pathway fidelity—rapid CT, physiology-led escalation, and IR as an adjunct to—not a replacement for—surgery—aligning our experience with contemporary guidance and large-institution series [14, 15]. These conclusions accord with multiphasic CT-driven strategies for detecting HVI and with governance models that emphasise timely escalation and standardised activation criteria [12, 21], and they are coherent with physiological rationales for organ-sparing haemostasis and preservation of hepatic perfusion [17–26].

## Conclusions

Under a protocolised, physiology- and CT-guided pathway at a university trauma centre, non-operative management was feasible for most hepatic injuries, with a low rate of delayed conversion to surgery, while selective angioembolisation served as an effective adjunct in appropriately selected patients. In this cohort, mortality appeared to be driven more by physiological derangement and overall injury burden than by hepatic injury grade alone, and the era-based analysis suggested improved system performance after formalisation of the management pathway, without evidence of a survival disadvantage among transferred patients. Taken together, these findings support a pathway-based non-operative strategy as the prevailing approach in liver trauma, while reserving surgery for haemodynamic instability, refractory haemorrhage, or associated injuries requiring laparotomy. These results, however, should be interpreted in light of several limitations. The retrospective single-centre design inherently exposes the study to selection bias, residual confounding, and dependence on the completeness and accuracy of recorded data. In addition, treatment allocation was not randomised, and the use of operative management or interventional radiology was necessarily shaped by clinical indication, thereby limiting causal inference. The findings may therefore not be fully generalisable to centres with different case mix, resource availability, or organisational structures. Future multicentre prospective studies are warranted to validate these observations in broader settings, to refine pragmatic bedside risk stratification models integrating anatomical grade with early physiological indices and overall injury severity, and to assess whether pathway adherence, including time to CT, access to interventional radiology, and predefined escalation thresholds, functions as a modifiable determinant of outcome.

## Data Availability

The dataset analysed in this study consists of de-identified routine clinical data collected at Policlinico Umberto I. The Institutional Review Board waived approval for this retrospective analysis but does not permit public release of individual-level data. De-identified data and a data dictionary are available from the corresponding author upon reasonable request, subject to a data-use agreement and institutional/GDPR approvals.
